# Mechanistic insights into subclinical *Plasmodium* infections: Unveiling the silent drivers of malaria transmission

**DOI:** 10.1016/j.isci.2026.115979

**Published:** 2026-05-17

**Authors:** Ritesh Ranjha, Aditi Gupta, Kuldeep Singh, Ruhi Sikka, Anup R. Anvikar, Himanshu Gupta, Praveen K. Bharti

**Affiliations:** 1 ICMR-National Institute of Malaria Research, New Delhi, Delhi, India; 2 Academy of Scientific and Innovative Research (AcSIR), Ghaziabad, Uttar Pradesh, India; 3 Department of Biotechnology, Institute of Applied Sciences & Humanities, GLA University, Mathura, Uttar Pradesh, India

**Keywords:** health sciences, clinical finding, disease

## Abstract

Subclinical malaria presents a challenge to global malaria elimination efforts. It often evades detection by conventional diagnostic tools due to low parasite densities. Despite being clinically silent, asymptomatic infections within the broader spectrum of subclinical malaria serve as hidden reservoirs that sustain transmission. Their persistence arises from a multifaceted interplay of parasite adaptations, including density thresholds, antigenic variation, cytoadhesion, multiplicity of infection, and emerging drug resistance, and host-related factors such as naturally acquired immunity, age, pregnancy, genetic polymorphisms, and epigenetic influences. Chronic subclinical and asymptomatic infections are associated with anemia, impaired cognitive development in children, and adverse pregnancy outcomes. This article examines the epidemiological burden, biological mechanisms, diagnostic challenges, and health consequences of subclinical malaria. The urgent need for field-deployable, sensitive diagnostic tools and integrative approaches is underscored. Understanding the mechanisms sustaining subclinical and asymptomatic infections is essential for targeted interventions and malaria elimination.

## Graphical abstract

**Figure:**
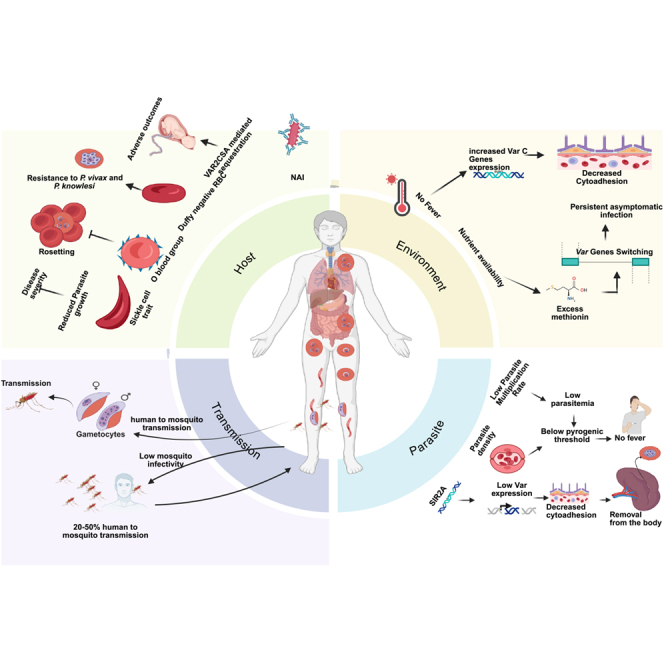

## Introduction

Malaria, caused by *Plasmodium* parasites, remains a global health challenge despite significant control efforts. While clinical malaria is characterized by fever, chills, and severe complications and primarily affects non-immune individuals such as young children and travelers, subclinical *Plasmodium* infections pose a silent yet critical barrier to malaria elimination.^1^ Subclinical malaria encompasses infections without overt febrile illness, which may be associated with measurable pathological consequences, including anemia, cognitive impairment, or immune modulation.^2^^,^^3^^,^^4^^,^^5^^,^^6^^,^^7^^,^^8^ Within this spectrum, asymptomatic malaria is defined as the presence of laboratory-confirmed parasitemia in individuals with no recent history of malaria-related symptoms or signs.^2^^,^^8^ These infections, driven by partial immunity from repeated exposures, allow parasites to persist at low densities, often undetected, yet capable of sustaining transmission.^9^^,^^10^

Globally, about 35 countries are pursuing malaria elimination, a goal hindered by these subclinical and asymptomatic reservoirs.^11^ Asymptomatic carriers, particularly school-age children and pregnant women, contribute 20–50% of human-to-mosquito transmission due to persistent gametocyte production, underscoring their epidemiological weight.^12^^,^^13^

The persistence of subclinical and asymptomatic malaria raises fundamental mechanistic questions. How do parasites evade host immunity without triggering symptoms? What host factors enable tolerance rather than clearance? How do environmental cues, such as seasonality or temperature, modulate these dynamics? Parasite strategies such as antigenic variation (e.g., via PfEMP1 in *P. falciparum*) and reduced cytoadhesion, coupled with host immune regulation (e.g., IL-10-mediated suppression), maintain low-density infections below pyrogenic thresholds.^14^^,^^15^^,^^16^ Epigenetic adaptations, including metabolic shifts (e.g., NAD+/NADH) and gene silencing (e.g., via SIR2a), further enable parasites to balance survival with transmissibility.^17^ These mechanisms, shaped by transmission intensity and host genetics, vary across *Plasmodium* species, with *P. falciparum* and *P. vivax* being the most studied.

Although subclinical malaria has been most extensively described in *P*. *falciparum* infections, increasing evidence indicates that non-P. *falciparum* species, including *P. vivax, P. ovale,* and *P. malariae*, also contribute to the subclinical parasite reservoir. Importantly, the biological basis of subclinical persistence differs across species. In *P. falciparum*, chronic subclinical infection is largely sustained by immune tolerance, antigenic variation, and low-density blood stage parasitaemia.^5^^,^^7^^,^^8^^,^^16^ In contrast, in *P. vivax* and *P. ovale*, prolonged infection duration may reflect both persistent blood stage infection and recurrent parasitemia arising from dormant liver stage hypnozoites.^5^^,^^18^^,^^19^

This review synthesizes the molecular and immunological underpinnings of subclinical malaria, with particular emphasis on asymptomatic infections within this spectrum, across parasite, host, and environmental factors. By dissecting how infections persist and fuel transmission, we aim to inform targeted strategies for elimination. The article explores: (1) the epidemiological scope, (2) parasite adaptations such as antigenic variation and density regulation, (3) host immunity and physiological modifiers, (4) environmental influences, (5) diagnostic challenges, (6) health consequences, and (7) control implications. Understanding these mechanisms is critical to developing precise diagnostics, therapies, and interventions to disrupt silent reservoirs and achieve a malaria-free world.

### Epidemiology of subclinical and asymptomatic *Plasmodium* infections

Approximately 60–90% of *Plasmodium* infections in high-endemic regions are subclinical, with a large proportion remaining asymptomatic.^20^^,^^21^^,^^22^^,^^23^ Even in low-transmission settings, up to 60% of infections occur without overt clinical symptoms.^24^ A study using Malaria Ag Pf/Pan RDTs reported that 15.2% and 18.8% of samples were positive for asymptomatic *P. falciparum*/Pan co-infections in high- and low-transmission areas of Tanzania, respectively.^25^ These low-density subclinical infections often go undetected and may contribute to sustaining local malaria transmission as hidden reservoirs.^20^^,^^26^

Most studies on subclinical or asymptomatic malaria have focused on *P. falciparum*, but there are increasing reports of asymptomatic infections caused by other species, including *P. vivax*, *P. ovale*, and *P. malariae*.^27^ Parasite densities are generally lower in *P. vivax* infections than in *P. falciparum* infections, as reported in high-endemic settings such as Papua New Guinea.^28^ Moreover, *P. vivax* has a lower pyrogenic threshold, defined as the parasite density at which fever develops, than *P. falciparum*.^29^ However, in malaria-endemic regions along the border between eastern Myanmar and northwestern Thailand, as well as in western Cambodia, no significant differences in parasite densities between *P. falciparum* and *P. vivax* have been observed in asymptomatic infections.^5^

Among individuals with subclinical infections, *P. falciparum* infections tend to persist longer than infections caused by other *Plasmodium* species. For instance, in Vietnam, 20% of *P. falciparum* (API = 25) and 4% of *P. vivax* (API = 17) infections were found to persist beyond four months.^30^ Interestingly, the median duration of asymptomatic infection was longer for *P. vivax* (60 days) than for *P. falciparum* (37 days) in low transmission settings, including Adama City, Oromia, Ethiopia.^31^

Subclinical parasite carriage may persist for extended periods. In rare cases, parasite carriage without symptoms has been documented for up to 13 years,^32^ with mechanisms likely differing between species, including prolonged blood-stage persistence in *P. falciparum* and recurrent parasitemia contributing to apparent long-term carriage in *P. vivax* and *P. ovale*. In Kassena-Nankana, a highly endemic district of Ghana, asymptomatic infections were observed to last as long as 179 days, although 21% of these infections cleared within one week. The duration was independent of age, suggesting that clearance may not be due to naturally acquired immunity (NAI) but rather to other innate mechanisms or genetic incompatibility between hosts and parasites.^33^^,^^34^^,^^35^ Multiclonal asymptomatic infections are more persistent than single-clone infections, likely due to greater immune evasion. The clearance rate for single-clone infections has been reported to be nine times faster than that for multiclonal infections.^36^^,^^37^ Residence in rural areas (high endemic) was associated with a 5-fold increase in the likelihood of subclinical parasite carriage compared with urban areas (moderate endemic) (*p* < 0.001) in the Democratic Republic of the Congo.^38^

A 20-year longitudinal study in Senegal demonstrated that *P. ovale* and *P. malariae* infections were largely asymptomatic, persisted at low parasite densities, and were associated with minimal morbidity, consistent with immune control without parasite clearance.^6^ However, *P. malariae* infections are also known to persist for prolonged periods if untreated and, in some cases, may lead to chronic complications such as nephrotic syndrome, indicating that apparently benign infections can still have significant long-term clinical consequences.^39^ The risk of fever in *P. ovale* and *P. malariae* infection increases in the presence of co-infection with other *Plasmodium* species. Similarly, a cohort study of Ugandan children showed that asymptomatic parasitemia increased the risk of subsequent clinical malaria, with only 11% of infections clearing without treatment, underscoring the clinical relevance of managing subclinical infections.^40^

Given their persistence and potential to sustain transmission, subclinical infections, including asymptomatic carriage caused by non-falciparum species, pose a significant challenge to malaria elimination efforts and call for a broader, non-falciparum species-inclusive approach to surveillance and control strategies. Recognizing the long-term carriage of multiple *Plasmodium* species highlights an urgent need to develop diagnostic tools and elimination strategies that can detect and clear low-density, mixed-species infections.

### Parasite factors

**Figure 1:**
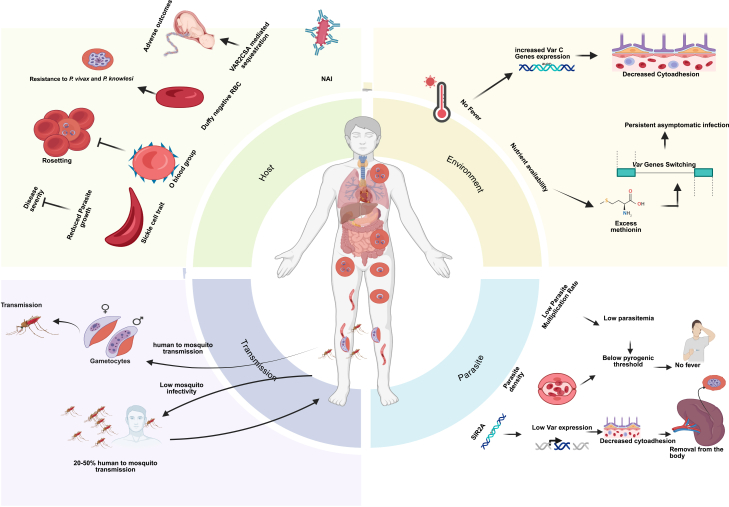
Factors contributing to asymptomatic malaria Multiple host and parasite determinants influence the persistence of *Plasmodium* infection without clinical symptoms. Naturally acquired immunity remains a key factor enabling parasite tolerance and controlling parasitemia below the pyrogenic threshold. Additional host factors—including the sickle cell trait, blood group O, and Duffy-negative red blood cells—further restrict parasite growth and reduce disease severity. In pregnancy, VAR2CSA-mediated sequestration of infected erythrocytes contributes to a substantial asymptomatic reservoir. Parasite adaptations such as altered *var* gene expression, reduced cytoadhesion, and lower multiplication rates help maintain low parasite densities, preventing febrile responses while allowing sustained low-level transmission (20–50% infectivity). Environmental modulators such as nutrient availability and temperature can further influence var gene switching and expression, promoting the establishment and maintenance of asymptomatic infections.

The malaria parasite employs several strategies to evade the host immune system and sustain infection, leading to diverse clinical outcomes.^41^ These include rapid multiplication, antigenic variation, cytoadhesion to host tissues, and hemozoin production. Antigenic variation allows the parasite to alter surface proteins, thereby avoiding immune recognition. Cytoadhesion and sequestration enable infected red blood cells (RBCs) to adhere to vascular endothelium, helping them escape splenic clearance. Asymptomatic carriage is often associated with low parasite densities and slower multiplication rates. Additionally, drug resistance can prolong infections by reducing the effectiveness of treatment. Together, these mechanisms facilitate long-term persistence of the parasite in individuals with subclinical infections, many of whom remain asymptomatic (Figure 1).

### Parasite density and pyrogenic threshold

The clinical severity of *P*. *falciparum* malaria is closely linked to the total parasite biomass, rather than circulating parasite density alone.^42^^,^^43^ The circulating *Plasmodium* density underestimates the total parasite biomass in severe cases of both *P. falciparum* and *P.vivax*.^42^^,^^44^ Subclinical malaria, including asymptomatic infections, is typically characterized by low parasite densities compared to clinical malaria,^45^ and the likelihood of developing fever increases with higher parasitemia levels, which is further influenced by the pyrogenic threshold.^46^ Notably, asymptomatic parasite densities tend to be lower in children than in adults,^5^ likely due to differences in naturally acquired, non-sterilizing immunity.^47^ This immunity develops over time through repeated exposures to the parasite, particularly in high-transmission settings.^47^

Transcriptomic studies have shown that the host’s inflammatory and immune gene expression is strongly influenced by parasite density. Low parasite burdens in individuals with subclinical infections may fail to trigger robust immune responses, contributing to the absence of clinical symptoms.^5^ One key immune evasion mechanism employed by *P. falciparum* is the antigenic variation of the PfEMP1 protein, mediated through the epigenetic regulation of *var* genes. This involves the *in situ* activation of a single *var* gene from a silenced repertoire, controlled by locus repositioning and chromatin remodeling. The NAD^+^-dependent histone deacetylase SIR2 plays a central role in this epigenetic silencing mechanism.^17^

Infection-induced lysis of RBCs releases parasite-derived metabolic by-products such as hemozoin and glycosylphosphatidylinositol (GPI), both of which are pro-inflammatory. These molecules are detected by pattern recognition receptors (PRRs) on innate immune cells such as macrophages, triggering the production of fever-inducing cytokines and other inflammatory mediators.^29^ As parasite density increases and surpasses the pyrogenic threshold, the accumulation of these pyrogens intensifies, often resulting in the onset of clinical symptoms. Importantly, the pyrogenic threshold is not fixed—it varies depending on factors such as the host’s age, transmission intensity in the region, parasite strain, and the individual’s immune status.^41^ Overall, the risk of fever and clinical disease increases with rising parasite density, with children typically exhibiting lower parasite thresholds than adults for symptomatic infections.^5^

Clarifying how host immunity and parasite epigenetic regulation maintain parasite densities below the febrile threshold may enable the identification of biological markers of subclinical infection, informing surveillance strategies that detect low-density infections before progression to symptomatic disease.

### Parasite multiplication rate

The parasite multiplication rate (PMR) reflects the fold increase or fold change in the number of blood-stage parasites over the course of one asexual cycle and is considered an approximate measure of parasite growth.^48^ This parameter is complexly regulated and has been linked to disease severity in malaria.^49^

In patients with severe malaria, the parasite multiplication rate has been observed to be up to three times higher than in those with uncomplicated malaria.^50^ Parasites in severe cases demonstrate a broader range of erythrocyte invasion—termed “unrestricted invasion”—compared to the more selective invasion patterns seen in mild or subclinical and asymptomatic infections.^50^ Additionally, both plasma PfHRP2 levels and peripheral parasitemia are significantly elevated in individuals with severe malaria, reinforcing their diagnostic and prognostic value.^49^

In asymptomatic malaria, parasite growth is tightly regulated through epigenetic mechanisms, including antigenic switching mediated by proteins such as sirtuins.^51^^,^^52^ In individuals with strong innate and adaptive immunity, parasitemia is maintained below symptomatic thresholds. Immune responses act broadly by limiting parasite invasion and replication, promoting clearance of infected erythrocytes, modulating inflammatory responses, and influencing var gene expression, thereby preventing disease progression.^51^^,^^53^ Specifically, the *P. falciparum* histone deacetylase Sir2a has been shown to regulate ribosomal DNA transcription, playing a key role in controlling the parasite’s replication rate.^53^

Host immune responses primarily act by clearing infected erythrocytes and parasite progeny rather than directly regulating parasite replication machinery.^54^ The combined effect of parasite-intrinsic growth control and immune-mediated clearance results in a low parasite multiplication rate, maintaining parasitemia below symptom-inducing thresholds.^5^^,^^55^

Recognizing how PMR shapes clinical outcomes highlights the need for studies that quantify epigenetic control of parasite growth in natural infections, which could inform new therapeutic or surveillance strategies.

### Antigenic variation

Diverse forms of PfEMP1 are encoded by the multicopy *var* gene family. The parasite expresses only one *var* gene at a time, and stochastic switching between these genes drives antigenic variation. This mechanism allows the parasite to evade the host immune response, contributing to prolonged, chronic infections that can persist for over a year.^15^^,^^16^^,^^56^ In malaria-endemic regions, repeated exposure to diverse PfEMP1 variants leads to the gradual development of broad antibody-mediated immunity, which helps control parasitemia and prevents severe disease.^15^^,^^16^^,^^56^^,^^57^

In asymptomatic infections, parasites tend to persist longer in circulation due to reduced cytoadhesion, leading to increased clearance by the spleen and consequently lower parasite densities.^58^^,^^59^^,^^60^ The var genes are broadly categorized into three major groups—A, B, and C—based on their chromosomal location and domain architecture. Their expression profiles are clinically relevant. Higher transcript levels of *var* group C are commonly associated with asymptomatic infections, whereas the elevated expression of *var* groups A and B has been linked to severe malaria.^61^ Group A PfEMP1 variants typically bind to endothelial protein C receptor (EPCR), while groups B and C bind to CD36.^62^^,^^63^

Interestingly, febrile temperatures in symptomatic malaria may alter the expression of SIR2a, a histone deacetylase involved in the epigenetic regulation of *var* gene expression. This temperature-dependent modulation may influence cytoadhesion properties and contribute to antigenic variation.^29^^,^^64^^,^^65^ *In vitro* studies have shown that heat shock and fever-associated host factors alter sirtuin and *var* transcriptional profiles, linking febrile responses to parasite epigenetic regulation. However, the establishment and persistence of chronic or asymptomatic infection primarily reflects a state of partial, non-sterilizing host immunity that constrains parasite density and limits pathology while allowing low-level survival and immune modulation.^64^

Asymptomatic infections are marked by the preferential expression of *var* group C genes, which are associated with weaker cytoadhesion and lower pathogenicity.^66^ Limited binding to endothelial receptors such as CD36 is associated with reduced sequestration and milder disease phenotypes characterized by low-density infections.^67^ Antigenic variation regulated by SIR2a facilitates immune evasion through controlled *var* gene expression, enabling chronic infection without overt clinical disease.^68^

Future work should clarify how environmental cues, such as fever and host immune pressure, fine-tune var gene switching, particularly the dominance of group C expression in asymptomatic infections. One plausible mechanism is that acquired humoral immunity against group A and B PfEMP1 variants selectively suppresses their expression, thereby skewing parasite populations toward the expression of less immunogenic group C variants. Understanding these regulatory and immune-driven triggers could inform strategies to disrupt chronic carriage and transmission.

### Cytoadhesion

In *P. falciparum* infections, infected erythrocytes adhere to endothelial cells via parasite-derived ligands that are transported to the RBC surface through specialized secretory structures known as Maurer’s clefts.^69^ These ligands are anchored within knob-like protrusions on the RBC membrane, facilitating firm adhesion to endothelial receptors.^70^ This cytoadhesion enables the sequestration of infected RBCs in the microvasculature, thereby avoiding splenic clearance—a mechanism unique to *P. falciparum* and central to its pathogenicity.^71^

The key cytoadhesive ligand responsible for this process is PfEMP1, which is expressed on the surface of infected RBCs and mediates binding to various host endothelial receptors.^72^ In addition to PfEMP1, other surface proteins, such as members of the repetitive interspersed family (RIFINs) also contribute to immune evasion and virulence. Both PfEMP1 and RIFINs undergo antigenic variation, allowing clonal populations of *P. falciparum* to evade host immunity by altering the surface-expressed antigenic profile, thereby avoiding antibody-mediated clearance.^72^ RNA polymerase III, involved in PfEMP1, is also downregulated in asymptomatic infections, leading to the decreased expression of PfEMP1.^73^ The cytoadhesion is reduced in asymptomatic infections due to the decreased expression of *var* genes. Laboratory studies have shown that parasites with markedly reduced or absent PfEMP1 expression exhibit substantially reduced cytoadhesion and are more readily cleared, resulting in lower parasite densities in experimental systems.^15^^,^^16^^,^^74^

In subclinical *P. falciparum* infections, particularly asymptomatic infections, reduced expression of *var* genes and downregulation of RNA polymerase III may lead to lower PfEMP1 levels, thereby limiting cytoadhesion and sequestration. This may further promote splenic clearance of infected erythrocytes and maintain low parasite densities.

Deciphering how parasites modulate PfEMP1 and other adhesins to balance survival with reduced sequestration may reveal new intervention targets. Insights into the regulatory pathways that suppress cytoadhesion in asymptomatic carriers may guide therapies aimed at curtailing transmission while minimizing disease severity.

### Multiplicity of infection

The genotype composition of *Plasmodium* parasites in the peripheral blood of individuals with subclinical infections can fluctuate daily. These temporal changes are believed to facilitate genotype selection and promote parasite survival during adverse conditions, such as the dry season.^30^ The peak multiplicity of infection (MOI)—defined as the number of distinct parasite clones present in an individual—typically occurs in children aged 3 to 7 years. In infants, parasite density is significantly correlated with MOI; however, this correlation becomes weak and non-significant in older children and adults.^75^

MOI tends to be higher in asymptomatic carriers residing in high-transmission settings compared to those in low-transmission areas.^76^ This pattern is likely due to repeated parasite exposure and the development of NAI, which allows individuals to tolerate multiple concurrent infections without developing symptoms.^76^

The relationship between MOI and the risk of developing symptomatic malaria remains inconclusive. Some studies suggest that individuals with multiclonal infections in high-transmission areas have a reduced risk of progressing to symptomatic malaria, possibly due to immune priming by diverse parasite strains.^77^^,^^78^^,^^79^ Conversely, other research has reported an increased risk of clinical malaria associated with multiclonal infections.^80^

Despite these uncertainties, asymptomatic infections with high MOI are known to contribute to malaria transmission, as they are capable of producing gametocytes—the transmissible stage of the parasite.^76^

Clarifying how fluctuating multiclonal infections interact with host immunity could illuminate why MOI sometimes protects against symptoms yet sustains transmission. Standardized, longitudinal genotyping studies are needed to resolve these conflicting associations and guide targeted interventions.

### Antimalarial resistance and subclinical malaria

Subclinical malaria infections, including asymptomatic infections, characterized by low-density parasitemia, serve as silent reservoirs for drug-resistant *Plasmodium* strains, complicating elimination efforts. Resistance markers, including mutations in dihydrofolate reductase (*pfdhfr*) and dihydropteroate synthase (*pfdhps*) genes, are prevalent in both asymptomatic and symptomatic infections across *Plasmodium* species, enabling sustained transmission of resistant parasites.^81^ In Myanmar, *P. falciparum* isolates from asymptomatic carriers showed high frequencies of resistance markers: *pfdhfr* (92.3%), *pfdhps* (97.6%), chloroquine resistance transporter (*pfcrt*, 84.0%), multidrug resistance protein 1 (*pfmdr1*, 98.8%), multidrug resistance-associated protein 1 (*pfmrp1*, 68.3%), and Kelch protein 13 (*k13*, 68.3%).^82^

The prevalence of resistance biomarkers in asymptomatic infections declines in low-transmission settings, where reduced drug pressure favors wild-type (drug-susceptible) parasites.^83^ Notably, resistance marker profiles in asymptomatic carriers mirror those in pre-treatment symptomatic cases, suggesting minimal selective pressure in asymptomatic infections.^37^

Mechanistically, asymptomatic infections sustain resistance by maintaining diverse parasite populations, including resistant strains, in the absence of clinical intervention. Multiclonal infections, common in high-transmission areas, may amplify this reservoir by increasing the likelihood of genetic recombination during the sexual stage of the parasite life cycle in the mosquito, potentially stabilizing resistance mutations.^84^ However, drug-susceptible parasites often outcompete resistant strains in low-transmission or therapy-free environments due to lower fitness costs, offering a strategic opportunity for control.^37^ Although exposure to antimalarial drugs has been found to increase the number of gametocytes in *Plasmodium* culture^85^ but no clear association has been found between antimalarial drug resistance and gametocyte carriage in patients with malaria.^86^ Effective mass drug administration (MDA) targeting symptomatic cases can reduce overall transmission, thereby limiting resistance spread, but must be paired with strategies to detect and treat asymptomatic reservoirs.^87^ For example, integrating ultrasensitive molecular diagnostics (e.g., PCR) to identify low-density resistant infections could guide targeted interventions, preserving antimalarial efficacy.^88^^,^^89^

Future research should examine how the competitive dynamics between drug-resistant and susceptible strains in low-transmission areas can be exploited to slow resistance spread, for example, by tailoring drug-rotation or MDA strategies to local fitness landscapes.

### Host factors in subclinical malaria

The clinical outcome of *Plasmodium* infections, ranging from symptomatic disease to subclinical and asymptomatic infections, depends on host factors such as age, immunity, pregnancy, and genetic traits (Figure 1). These factors shape the host’s ability to tolerate low-density infections, sustaining transmission reservoirs critical to malaria’s persistence.

### Naturally acquired immunity

Repeated exposure to malaria parasites early in life leads to the development of NAI against clinical malaria.^20^^,^^26^^,^^47^ Due to NAI, individuals can harbor malaria parasites without displaying acute febrile illness.^20^^,^^27^ This immunity reduces disease severity and contributes to asymptomatic infections.^27^ Remarkably, NAI against clinical malaria includes both innate and adaptive immune responses.^34^ It can develop only after one or two infective mosquito bites, highlighting the importance of strain-specific immunity, where immunity is effective primarily against the particular parasite strains previously encountered, but not others.^27^^,^^34^ Although NAI controls clinical symptoms, it does not usually clear the parasite; clearance may instead result from other innate immune mechanisms or genetic incompatibilities between host and parasite.^20^^,^^33^

With repeated *P*. *falciparum* exposure, the immune system adapts by generating regulatory responses dependent on exposure and parasite specificity. These responses involve decreased production of pro-inflammatory cytokines (IL-1β, IL-6, IL-8) and increased anti-inflammatory cytokines such as IL-10 and TGF-β. This immunological balance enhances phagocytic activity and activates adaptive immunity, reducing harmful inflammation while effectively controlling parasites.^90^ Elevated IgE levels in subclinical *P. falciparum* infections, particularly asymptomatic infections, are also associated with decreased risk of symptomatic malaria.^91^

Both adaptive and innate immune responses contribute to asymptomatic malaria.^92^ Controlled inflammation and antibody production are crucial.^93^ Innate immune PRRs detect parasite molecules such as hemozoin, GPI, and AT-rich or CpG DNA motifs bound to hemozoin.^93^^,^^94^ PRR activation induces cytokines and chemokines that inhibit parasite growth and shape adaptive immunity.^14^ Strong antibody responses against antigens such as AMA-1, MSP-1, and MSP-3 correlate with protection from symptomatic malaria.^95^ Protein microarray studies have identified antibodies to over 100 *Plasmodium* antigens—including MSP2, MASP7, MSP10, liver-stage antigen 3, Pf70, erythrocyte membrane protein 1, and helical interspersed subtelomeric domain protein—that predict protection against symptomatic disease in children.^96^

Asymptomatic individuals typically have higher levels of *P. falciparum*-specific IgG antibodies, reflecting a robust humoral response that influences clinical outcomes.^92^ Antibody levels increase incrementally with each exposure until young adulthood, contributing to gradual immunity acquisition.^14^ High neutrophil antibody-dependent respiratory burst (ADRB) activity and elevated expression of human leukocyte antigen-DR on dendritic cells also correlate with subclinical infection.^92^

Notably, the absence of pro-inflammatory TNF-producing CD4^+^ T cells is linked to asymptomatic infections, suggesting that IL-10 production by T-helper cells may prevent symptomatic malaria.^92^ Loss of IL-10 upregulation capacity has been observed in T cells from children no longer exposed to malaria, indicating the importance of ongoing exposure in maintaining regulatory responses.^14^

NAI, developed through repeated parasite exposure, enables asymptomatic *P. falciparum* infections by modulating immune responses and controlling parasite densities below pathology-inducing thresholds. The low pro-inflammatory and elevated anti-inflammatory signals (e.g., IL-10 and TGF-β), limit pathology while limiting parasitemia at subclinical levels. Controlled inflammation, a loss of IL-10 upregulating capacity in T cells, and a strong antibody response against multiple *Plasmodium* antigens contribute to protection against symptomatic disease.

Future work should dissect how long NAI persists after exposure ends and which specific B- and T cell memory compartments sustain protection. Such insights could guide vaccine designs that mimic naturally acquired immune profiles without requiring repeated infections.

While NAI plays a dominant role in controlling parasitemia and clinical symptoms in *P. falciparum* infections, its contribution to subclinical infections patterns in *P. vivax* is less clearly defined, owing to the confounding influence of hypnozoite-driven relapses.

### Influence of age

Parasite densities in subclinical infections, particularly asymptomatic infections are generally lower in children than adults,^5^ and infections tend to be shorter in older individuals regardless of *Plasmodium* species.^97^ In moderate to high transmission areas, children over three years old with asymptomatic parasitemia have a reduced risk of febrile malaria. However, in low-transmission settings, asymptomatic parasitemia increases the risk of febrile malaria across all pediatric age groups.^98^ Although malaria affects all ages, clinical risk decreases with age.^99^ Studies show that asymptomatic parasite prevalence declines with increasing age among adults aged 15–54, peaking in the 15–25 age group.^100^ School-aged children (6–14 years) serve as significant reservoirs of asymptomatic malaria in endemic regions.^20^^,^^101^ Children under five, with immature immune systems, are more vulnerable to malaria morbidity and mortality, while older children often develop clinical immunity and have a higher prevalence of asymptomatic infections.^26^

Age and parasite exposure shape asymptomatic malaria. Young children have lower parasite densities but higher clinical risk, while older children and adults develop partial immunity, enabling asymptomatic carriage. Longitudinal cohort studies that track individuals from infancy through adulthood could clarify how age-related immune maturation interacts with transmission intensity to shape asymptomatic carriage. Such data would help refine age-targeted surveillance and intervention strategies, particularly for school-aged children who remain key hidden reservoirs.

### Subclinical malaria in pregnancy

Pregnant women are more susceptible to malaria due to immune modulation and physiological changes. During pregnancy, *P. falciparum*-infected erythrocytes express the variant surface antigen VAR2CSA, which mediates adhesion and sequestration in the placenta. This interaction triggers placental inflammation and disrupts normal development, adversely affecting fetal growth.^102^

Primigravid women (first pregnancy) are at higher risk of adverse fetal outcomes from malaria. Subsequent pregnancies typically show decreased infection intensity, likely due to antibody development against variant surface antigens.^27^ In areas of stable malaria transmission, most *Plasmodium* infections during pregnancy are subclinical and asymptomatic.^103^ A meta-analysis in Ethiopia found a high pooled prevalence of asymptomatic malaria in pregnant women, associated with stagnant water and insecticide-treated net usage.^104^ Anemia prevalence is also higher among pregnant women with asymptomatic malaria.^105^ Elevated plasma levels of IL-10 and G-CSF in subclinical malaria during pregnancy may contribute to susceptibility to symptomatic disease.^106^

Pregnancy increases malaria susceptibility through immune modulation and placental sequestration via VAR2CSA. Primigravidae lack protective antibodies, heightening risk, with decreased risk in subsequent pregnancies. Asymptomatic infections are common in stable transmission areas, contributing to anemia and adverse outcomes. Elevated IL-10 and G-CSF in asymptomatic malaria in pregnancy may impair effective immune clearance, increasing vulnerability to clinical malaria.

Asymptomatic malaria in pregnancy highlights a dual challenge: protecting maternal-fetal health while addressing an under-recognized transmission reservoir. Future research should investigate how VAR2CSA-targeted immunity evolves across successive pregnancies and whether novel vaccines or monoclonal antibodies can provide early protection for primigravidae. Integrating highly sensitive screening (e.g., PCR-based tests) into routine antenatal care, especially in low-resource, moderate-transmission settings, could enable timely treatment and reduce placental sequestration-related complications.

### Genetic factors

Host genetic factors are key determinants in disease presentation and pathogenesis.^107^ Host genetic traits modulate the course of subclinical malaria. While some studies suggest a possible role of ABO blood group in influencing parasitemia, findings remain inconsistent, and a meta-analysis reported no clear association.^108^^,^^109^^,^^110^^,^^111^ By contrast, the Duffy blood group antigen is critical for *P. vivax* invasion, and Duffy negativity confers resistance, often resulting in low-density asymptomatic infections detectable only by molecular tools.^112^^,^^113^^,^^114^ Hemoglobinopathies such as sickle cell trait and α^+^-thalassemia, protect against severe malaria but show variable effects on asymptomatic carriage.^115^^,^^116^^,^^117^ G6PD deficiency has also been linked to reduced risk of asymptomatic *P. falciparum* infection, with specific variants (e.g., 202A/376G and G6PD A–) associated with protection in some populations.^3^^,^^118^^,^^119^ Overall, genetic polymorphisms shape host susceptibility and infection dynamics, but their role in sustaining subclinical malaria remains incompletely understood.

Host genetic diversity provides valuable clues to malaria pathogenesis, yet its impact on asymptomatic carriage is still poorly defined. Large, multi-ethnic genomic studies integrating parasite genotyping with longitudinal clinical data could clarify how variants such as G6PD deficiency or hemoglobinopathies influence parasite persistence and transmission potential. Such insights may guide personalized interventions and improve risk stratification in elimination programs.

### Environmental and epigenetic influences

Subclinical malaria, including asymptomatic infections, is shaped by both environmental and host factors (Figure 1). In high-transmission regions, NAI supports multiclonal, chronic infections, while even in low-transmission settings, a substantial proportion of infections remain subclinical, complicating elimination.^1^^,^^10^^,^^120^ Parasite survival at low densities is partly mediated by the epigenetic regulation of *var* genes, which encode PfEMP1 proteins involved in cytoadherence and immune evasion. This regulation is influenced by sirtuins (PfSIR2A/B) and modulated by host stressors such as fever and nutrient availability, promoting antigenic variation.^64^^,^^66^^,^^121^^,^^122^^,^^123^ Seasonality further shapes subclinical malaria, with prevalence persisting year-round, even when symptomatic cases decline during dry seasons, likely due to splenic clearance maintaining low-density infections.^20^^,^^26^^,^^58^^,^^124^ Seasonal changes in host plasma composition have little effect on parasite growth or survival, whereas the persistence of low-density parasitemia during the low-transmission season depends on the timing of infection, with clones transmitted late in the wet season showing a greater likelihood of surviving the dry season.^59^ Together, environmental pressures and epigenetic plasticity enable parasites to persist silently, sustaining transmission reservoirs. These epigenetic mechanisms are most relevant to *P. falciparum*, whereas in *P. vivax* and *P. ovale*, environmental persistence is additionally shaped by liver-stage hypnozoites that operate independently of blood-stage immune and epigenetic regulation.

Understanding how environmental pressures interact with parasite epigenetic programs is crucial for malaria control. Integrated studies linking climate variability, host metabolic cues, and var-gene regulation could reveal trigger points for parasite recrudescence and help identify seasonal windows where targeted interventions would most effectively disrupt silent transmission.

### Subclinical malaria and mosquito infection

Gametocytes are the only life stage of the malaria parasite capable of being transmitted from humans to mosquitoes. In *P. falciparum* infections, gametocytes often constitute about 5% of the total parasite biomass within the human host. Successful transmission from humans to mosquitoes depends on multiple factors, including gametocyte density, the host’s immune response, local transmission intensity, and the mosquito’s susceptibility to infection.^125^

Gametocyte carriage remains high even in individuals with afebrile parasitemia. Approximately 70% of subclinical infections have been reported to be gametocyte-positive.^125^ However, at sub-microscopic gametocyte densities—those undetectable by light microscopy—mosquito infection rates are two-to 5-fold lower compared to densities detectable by microscopy.^126^ Notably, mosquito infection rates rise sharply when gametocyte density exceeds 100–200 parasites/μL of blood.^94^

Among individuals with symptomatic malaria, those with microscopy-positive blood smears are 20 times more infectious to mosquitoes than smear-negative individuals.^127^ Interestingly, in a study conducted in Ethiopia, mosquito infection rates were found to correlate with asexual parasite density in *P. vivax* infections, but not in *P. falciparum* infections.^13^ This difference likely reflects fundamental disparities in gametocyte development time between the two species. In *P. vivax*, gametocytes are produced rapidly within approximately 48 h, resulting in a closer temporal association between asexual parasitemia and transmissible sexual stages. In contrast, the 9 to 12 days maturation period of *P. falciparum* gametocytes allows substantial discordance between asexual parasite density and infectiousness.^128^

In mosquito feeding studies, subclinical *P. falciparum* infections were infectious in 6.4% of cases, while asymptomatic *P. vivax* infections did not result in mosquito infections. However, a mixed-species infection (*P. falciparum* and *P. vivax*) was found to be highly infectious.^31^ The low mosquito infectivity of asymptomatic individuals has been attributed to their low parasite and gametocyte densities.^31^

Despite their lower individual infectivity, asymptomatic infections within the broader subclinical reservoir represent a major source of malaria transmission due to their high prevalence in endemic areas.^20^^,^^97^ Asymptomatic carriers are estimated to be responsible for 20–50% of all human-to-mosquito transmission events, with the likelihood of transmission increasing alongside gametocyte density,^12^^,^^13^ highlighting the need to target asymptomatic infections in elimination strategies.

### Subclinical malaria and the diagnostic dilemma

Subclinical malaria often involves low peripheral parasitemia but relatively high gametocyte densities, sustaining transmission despite of overt febrile illness.^125^ Standard diagnostics—RDTs and microscopy—miss many infections: a Ghana study found ∼40% undetected,^129^ and a meta-analysis showed only 48.7% of PCR-positive infections detected by microscopy and 4.8% by RDTs.^130^^,^^131^ Different diagnostic methods have varying limits of detection, sensitivities, and specificities, as shown in Table 1.

**Table 1:** Malaria diagnostic methods with their sensitivity and specificity

Diagnostic Method	Sensitivity	Specificity	Time Required	Feasibility	Limitations	Reference
RDT	moderate (detects >200 parasites/μL)	high (≈95–99%)	15–30 min	easy to use; suitable for field use; no equipment needed	limited sensitivity for low parasitemia and asymptomatic infections, false negatives with *pfhrp2/pfhrp3* deletions	Cunningham et al. and Hofmann et al^132^^,^^133^
Ultra-sensitive RDT	high (detects ∼10 parasites/μL)	high (≈95–98%)	15–30 min	field-feasible; similar procedure as RDT, may be useful for asymptomatic and low-density infections	slightly costlier; affected by *pfhrp2* deletions, limited availability	Hofmann et al and Landier et al^133^^,^^134^
Microscopy	moderate to high (50–100 parasites/μL depending on skill)	high (>95%)	30–60 min	feasible, widely available, low cost	requires skilled microscopist, poor detection of low-density or mixed infections	WHO et al., and Wongsrichanalai et al^135^^,^^136^
PCR	very high (<1 parasite/μL)	very high (>99%)	3–6 h	not feasible, ideal for research, good for detecting asymptomatic infections	expensive, requires lab setup and expertise,	WHO et al., and Oyedeji et al^137^^,^^138^
Real-time PCR	very high (0.1 parasites/μL)	high (>95%)	1.5–2.5 Hrs	not feasible, ideal for research, highly sensitivity to detect asymptomatic infections	expensive, requires lab setup and expertise, Costly consumables	Taylor et al., and Lazrek et al^139^^,^^140^
Loop-mediated isothermal amplification test (LAMP)	very high (<1 parasite/μL)	high (>95%)	15-60 min	no special equipment required, less time required than PCR	cold chain requirement for reagents, requires a basic DNA isolation setup, and a lack of uniform protocol	Fitri et al., and Ivarsson et al^141^^,^^142^

HRP2-based RDTs, the primary tool for *P. falciparum* detection,^143^ show variable sensitivity with age, parasite density, transmission intensity, and prevalence.^131^^,^^143^^,^^144^ Although useful in field settings, they often fail to identify subclinical carriers and are further compromised by *pfhrp2* gene deletions.^143^^,^^145^^,^^146^ Ultra-sensitive HRP2 RDTs match or exceed microscopy in research studies but require validation in national programs.^145^^,^^146^

Highly sensitive molecular methods—nested PCR, multiplex/real-time PCR, and LAMP—detect as few as 0.1–6 parasites/μL^147^^,^^148^ but need specialized equipment, skilled staff, and longer turnaround, limiting field use. Closing these diagnostic gaps is essential to identify hidden reservoirs and achieve malaria elimination.

### Other health consequences of subclinical and asymptomatic malaria infections

In addition to serving as a hidden reservoir for malaria transmission, subclinical malaria infections, including asymptomatic infections, have significant health consequences, particularly in children. School-age children are consistently identified as the primary carriers of subclinical *Plasmodium* infections across various transmission settings.^13^^,^^20^^,^^26^^,^^149^ These infections are associated with a higher risk of anemia,^4^ and growing evidence suggests that chronic parasitemia negatively impacts cognitive development and school performance.^101^^,^^150^^,^^151^^,^^152^^,^^153^ Non-severe *Plasmodium* infections have been associated with poorer school performance in the Brazilian Amazon.^150^ In high transmission settings, subclinical *Plasmodium* infections were strongly associated with poorer performance in tests of abstract reasoning and sustained attention.^151^ The effect of subclinical infection on cognitive development may be mediated through subclinical inflammation in the asymptomatic children.^152^

Thrombocytopenia is another health effect linked to persistent *P. falciparum* infections. Parasites are thought to promote infection persistence by downregulating platelet counts in the host.^153^ Moreover, subclinical *P. falciparum* infection has been implicated in the etiology of endemic Burkitt’s lymphoma (eBL) in African populations. The similar mean age of children with asymptomatic malaria and those diagnosed with eBL suggests a potential link, with persistent subclinical parasitemia possibly increasing the risk of lymphoma development.^153^^,^^154^

Due to the immune modulation in subclinical malaria infections, the risk of invasive bacterial infections is increased. Chronic low-density infections with accompanying hemolysis may cause neutrophil dysfunction contributing to invasive bacterial infections and mortality.^1^

These adverse outcomes, likely mediated by chronic low-grade inflammation, highlight the need to address subclinical malaria not only for its role in transmission but also for its direct impact on individual health, particularly among vulnerable pediatric populations. Subclinical malaria is frequently associated with chronic low hemoglobin levels. The presence of background anemia increases the risk of progression to severe malaria, with the highest risk observed in children with severe anemia (hemoglobin <5 g/dL).^155^ Co-occurrence of *Plasmodium* infection and anemia has been associated with a 3.5-fold increase in mortality risk.^156^ Moreover, in the context of co-infections such as tuberculosis, anemia linked to subclinical malaria may increase the risk of disease recurrence 2-fold and mortality by two-to 3-fold.^157^

Subclinical malaria infections, including asymptomatic infections, are therefore associated with recurrent parasitemia, chronic anemia, maternal and neonatal mortality, increased susceptibility to invasive bacterial diseases, cognitive impairment, and sustained parasite transmission. Given these cumulative health consequences, such infections are more appropriately referred to as “chronic” malaria infections rather than merely a silent reservoir.^1^

### Implications for malaria control and elimination

Subclinical malaria infections, including asymptomatic infections, were historically left untreated due to their lack of clinical symptoms. However, recent evidence highlights their critical role in sustaining malaria transmission, even during low-transmission periods.^97^ These persistent infections can bridge transmission across seasons and pose a major obstacle to achieving malaria elimination targets, especially in countries aiming for elimination.^26^^,^^97^

In high-transmission settings, repeated exposure leads to the development of NAI, resulting in a high prevalence of subclinical infections. Conversely, in low-transmission regions, transmission tends to cluster in specific hotspots—often characterized by a high burden of subclinical carriers—that perpetuate local malaria transmission.^158^

School-age children have emerged as a key reservoir for subclinical *Plasmodium* infections. Despite their importance, this group is generally not targeted by existing malaria control strategies. Focused interventions in this demographic could significantly accelerate progress toward elimination goals.^20^^,^^26^

Drug-based strategies aimed at clearing subclinical infections include MDA, mass screening and treatment (MSaT), and focal screening and treatment (FSaT). Among these, MDA is particularly effective at temporarily reducing the asymptomatic parasite reservoir but shows only a short-term impact in high-transmission areas. In contrast, it can be more impactful in low-transmission settings, particularly when combined with strong vector control measures. However, the World Health Organization recommends MDA primarily for isolated settings such as islands or pre-elimination zones with minimal risk of reintroduction.^159^

Control strategies must account for species-specific drivers of subclinical malaria. While interventions targeting chronic blood-stage infections are critical for *P. falciparum*, elimination of *P. vivax* and *P. ovale* requires concurrent targeting of hypnozoite reservoirs to prevent relapse-driven subclinical carriage.

MSaT using highly sensitive diagnostics is another promising approach. MSaT can contribute to malaria elimination by identifying and treating low-density infections that would otherwise go undetected.^160^ However, its success hinges on the availability of field-deployable diagnostic tools with low detection thresholds. The development and deployment of such point-of-care molecular diagnostics is essential for effectively targeting the hidden reservoir of asymptomatic infections and advancing malaria elimination efforts.^161^

The World Health Organization Global Technical Strategy for Malaria recommends integrated vector control, chemoprevention, and prompt diagnostic testing and treatment to reduce transmission and prevent malaria-associated morbidity and mortality. Currently recommended diagnostic tools include microscopy and antigen-based rapid diagnostic tests (RDTs).^162^^,^^163^^,^^164^^,^^165^ Countries such as El-Salvador, Sri Lanka, and China have achieved malaria elimination by implementing WHO-defined elimination frameworks supported by strong political commitment, robust surveillance systems, and locally tailored intervention strategies. In particular, China adopted the “1-3-7” strategy for case reporting, investigation, and response, which operationalized WHO elimination criteria through rapid surveillance and targeted action.^163^^,^^164^^,^^165^

**Figure 2:**
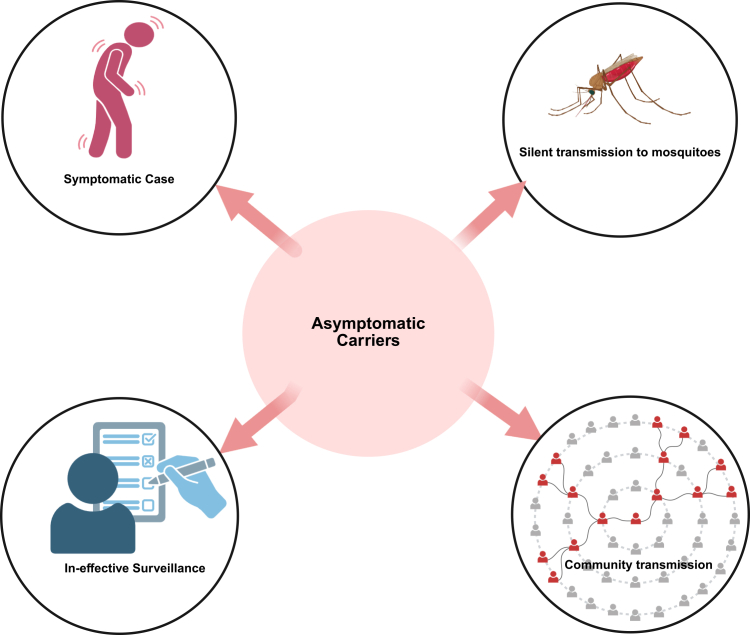
Asymptomatic carriers and malaria control Asymptomatic carriers can undermine surveillance efforts, as they sustain malaria transmission silently and may later develop symptoms, thereby reducing the overall effectiveness of case detection and control programs.

Despite these successes, subclinical infections sustain malaria transmission by bridging low-transmission periods and forming local hotspots (Figure 2). A cohort study of Ugandan children found that 74% of subclinical parasitemia progressed to subsequent clinical malaria and only 11% cleared without treatment, indicating the significance of treating the asymptomatic infections.^40^ The use of highly sensitive diagnostic tools has demonstrated that the prevalence of asymptomatic malaria is substantially higher than previously recognized, enabling the detection of hidden community reservoirs.^97^ Therefore, if malaria elimination is a priority, subclinical and asymptomatic malaria can no longer be overlooked.^97^

Clearing asymptomatic infections via MDA or MSaT can help in elimination, but success depends on combining it with highly sensitive, field-ready diagnostics to detect low-density infections.^166^ Effective elimination requires shifting focus from symptomatic cases alone to include subclinical carriers, particularly asymptomatic individuals. Prioritizing school-age children and integrating sensitive diagnostics with targeted interventions can close transmission gaps and accelerate progress toward malaria elimination.

### Conclusion

Due to its silent clinical profile, subclinical malaria, including asymptomatic infections, poses a major challenge to malaria elimination efforts. These infections act as hidden reservoirs that silently sustain transmission within communities. Subclinical malaria results from a complex interplay between parasite, host, and environmental factors (Figure 1), with species-specific mechanisms including chronic blood-stage persistence in *P. falciparum* and hypnozoite-mediated relapse in *P. vivax* and *P. ovale* shaping long-term carriage and transmission. Mechanistically, the parasite employs immune evasion strategies, such as antigenic variation and reduced cytoadhesion, to persist in the host without triggering acute symptoms. On the host side, immune tolerance, shaped by NAI and regulatory cytokines such as IL-10, allows the parasite to remain without inducing a strong inflammatory response. Environmental factors, including epigenetic adaptations and seasonal transmission dynamics, further modulate the occurrence and persistence of subclinical carriage (Figure 2). Untreated subclinical infections may compromise effective surveillance due to continued transmission.

Effectively addressing subclinical malaria will require a combination of sensitive diagnostic tools, targeted interventions, and strengthened programmatic frameworks. Routine integration of subclinical malaria detection into national elimination programs is essential to identify and treat hidden infections that sustain transmission. The development and deployment of affordable, ultra-sensitive diagnostic assays should be prioritized to enable the detection of low-density infections in field settings. Furthermore, the evaluation of targeted MDA and reactive screening strategies can help interrupt local transmission foci. Strong investment in surveillance systems is required, particularly in countries approaching or in the elimination phase. It will be critical to monitor residual transmission and prevent resurgence.

An integrated approach that builds on existing control measures, enhances surveillance, and incorporates technological and operational innovations will be key to identifying and eliminating subclinical infections, including asymptomatic carriers, thereby accelerating progress toward global malaria elimination targets.

## Acknowledgments

The authors would like to acknowledge ICMR-National Institute of Malaria Research and GLA University, Mathura, for logistical support.

Funding: No funding was received for this work from any agency or organization.

## Author contributions

R.R., H.G., A.R.A., and P.K.B. designed and conceptualized the study; R.R., A.G., K.S., and R.S. collected the literature and wrote the first draft of the manuscript; H.G., A.R.A., and P.K.B. critically reviewed the manuscript. All authors read and approved the final version of the manuscript.

## Declaration of interests

No potential conflict of interest was reported by the authors.

## Declaration of generative AI and AI-assisted technologies in the writing process

During the preparation of this work, the author(s) used ChatGPT to improve the language and readability. After using this tool, the author(s) reviewed and edited the content as needed and take full responsibility for the content of the published article.
